# Characterizing upper extremity motor behavior in the first week after stroke

**DOI:** 10.1371/journal.pone.0221668

**Published:** 2020-08-10

**Authors:** Jessica Barth, Shashwati Geed, Abigail Mitchell, Peter S. Lum, Dorothy F. Edwards, Alexander W. Dromerick

**Affiliations:** 1 MedStar National Rehabilitation Network, Washington, District of Columbia, United States of America; 2 Department of Rehabilitation Medicine, Georgetown University, Washington, District of Columbia, United States of America; 3 Department of Biomedical Engineering, Catholic University of America, Washington, District of Columbia, United States of America; 4 Department of Kinesiology and Occupational Therapy, University of Wisconsin, Madison, Wisconsin, United States of America; University of Ottawa, CANADA

## Abstract

**Background:**

Animal models of brain recovery identify the first days after lesioning as a time of great flux in sensorimotor function and physiology. After rodent motor system lesioning, daily skill training in the less affected forelimb reduces skill acquisition in the more affected forelimb. We asked whether spontaneous human motor behaviors of the less affected upper extremity (UE) early after stroke resemble the animal training model, with the potential to suppress clinical recovery.

**Methods:**

This prospective observational study used a convenience sample of patients (n = 25, mean 4.5 ±1.8) days after stroke with a wide severity range; Controls were hospitalized for non-neurological conditions (n = 12). Outcome measures were Accelerometry, Upper-Extremity Fugl-Meyer (UEFM), Action Research Arm Test (ARAT), Shoulder Abduction/ Finger Extension Test (SAFE), NIH Stroke Scale (NIHSS).

**Results:**

Accelerometry indicated total paretic UE movement was reduced compared to controls, primarily due to a 44% reduction of bilateral UE use. Unilateral paretic movement was unchanged. Thus, movement shifted early after stroke; bilateral use was reduced and unilateral use of the non-paretic UE was increased by 77%. Low correlations between movement time and motor performance prompted an exploratory factor analysis (EFA) revealing a 2-component solution; motor performance tests load on one component (motor performance) whereas accelerometry-derived variables load on a second orthogonal component (quantity of movement).

**Conclusions:**

Early after stroke, spontaneous overall UE movement is reduced, and movement shifts to unilateral use of the non-paretic UE. Two mechanisms that could influence motor recovery may already be in place 4.5 ± 1.8 days post stroke: (1) the overuse of the less affected UE, which could set the stage for learned non-use and (2) skill acquisition in the non-paretic limb that could impede recovery. Accurate UE motor assessment requires two independent constructs: motor performance and quantity of movement. These findings provide opportunities and measurement methods for studies to develop new behaviorally-based stroke recovery treatments that begin early after onset.

## Introduction

Of the estimated 7 million stroke survivors in the United States, up to 88% are thought to have upper extremity (UE) motor involvement [1–3]. This motor impairment is usually disabling, leading to the need for modification or assistance in activities of daily living (ADL) and reduced social participation [4–6].

Several motor behavioral factors (timing of motor training, dosing, and use of non-paretic forelimb) and various medications meaningfully influence rodent forelimb motor recovery [7–9]. These factors also seem relevant to conventional human rehabilitation in clinical settings, with the potential to influence final motor outcomes in patients. In rodents, starting five days after motor cortex lesioning, twenty minutes of daily skill training of the less affected forelimb has been shown to reduce skill acquisition in the more affected forelimb [10, 11]. If there are parallels in human clinical populations, training compensation skills (in formal therapy or informally by a patient or family) in the non-paretic limb might conceivably inhibit motor recovery or facilitate learned non-use [12–17].

Given the potential for these motor behaviors and interventions to affect motor recovery, systematic study of the human motor system in the first days after stroke is essential. In order to prepare for more detailed and hypothesis driven studies, we undertook a preliminary cross-sectional study to identify and characterize the range of UE motor behaviors during the first week after stroke, using widely accepted clinical measures and also objective quantification using wrist-worn accelerometers. We hypothesized that measures of motor impairment would be highly correlated, and that the amount of spontaneous movement (whether random, goal-directed, passive, or active) would be tightly linked to motor severity.

## Methods

### Participants

The study was approved by the MedStar Health IRB. In this preliminary work, we enrolled a convenience sample with a wide variety of stroke severity, capturing a range of motor behaviors. Study participants were identified via screening logs maintained by the Stroke Central Atlantic Network for Research [18]. A total of 448 patients were screened via electronic medical health record from MedStar Washington Hospital Center from April 16, 2017 to May 11, 2018; participants were consented ≤ 7 days of onset from the acute stroke service. A total of 96 were not available for evaluation for clinical reasons, 91 were discharged before evaluation, 88 were outside the time window, 39 had other neurologic conditions, 28 were enrolled in another study and 26 had prior stroke which was unrecovered. The remainder were excluded for a variety of other reasons (not expected to be available at 30 days, bilateral stroke, etc.). All participants had imaging-confirmed unilateral ischemic or hemorrhagic stroke. Participants were excluded if they had a history of prior stroke with residual UE weakness, prior relevant orthopedic or neurological conditions that limited or potentially altered UE movement, and enrollment in a conflicting clinical trial. To control for non-specific UE use in hospitalized individuals, we recruited a second cohort of adult inpatients (n = 12) with non-neurological conditions.

### Measures

*The Upper Extremity Fugl-Meyer* (UEFM) assesses motor impairment at the shoulder, elbow, wrist and fingers along with passive range of motion (PROM), pain and sensation. A full score is 66 with higher scores reflecting better motor function [19, 20].

*The Action Research Arm Test* (ARAT) assesses UE functional limitation. The ARAT uses a 4-point ordinal scale on 19 items to measure grasp, grip, pinch and gross motor movements of both UE [21–23].

*The Shoulder Abduction- Finger Extension* (SAFE) strength assessment sums manual muscle testing of shoulder abduction and finger extension to produce a score from 0–10 [24–26]. A score of 10 indicates full strength in both movements.

*Goniometry* was performed according to standard methods at the wrist, fingers and elbow [27].

*The National Institutes of Health Stroke Scale* (NIHSS), a standardized neurological assessment, describes overall stroke severity. The UE motor item scores the participant on their ability to maintain shoulder flexion at 90 degrees and full antigravity extension of other UE joints for 5 seconds. Scores range from 0 (no impairment) to 4 (no movement) [28, 29].

The *Mesulam Symbol Cancellation Test* (unstructured condition) screens for visuospatial neglect [30]. This measure involves identifying visual targets within a page of distractors; asymmetry of >3 errors indicates neglect.

*The Frenchay Aphasia Screening Test* (FAST) is a brief screening test for aphasia after stroke [31]. The full scale is composed of four subscales; a higher score indicates more language impairment.

*The Wong-Baker Pain Rating Scale* assesses pain [32, 33].

*Accelerometry* (Actigraph GT9X Link Activity Monitor; Actigraph, Pensacola, FL) was used as an objective and quantitative measure of UE use bilaterally. Accelerations were recorded along 3 axes at 50Hz [34, 35]. Testing in our laboratory showed that the devices detected movements as small as 0.5cm (unpublished data).

### Procedures

All participants or their proxy provided written informed consent and received standard acute stroke clinical care. A licensed Occupational Therapist performed all clinical assessments following accelerometer placement to both wrists. Participants and/or their proxy were educated on the 24-hour wear schedule of the accelerometers, and signage was placed in the room with this information. Similar education was provided to the clinical staff. Accelerometers were applied bilaterally just above the ulnar styli for at least 24 hours; data were normalized to 24 hours’ wear.

### Analysis

Participants and clinical staff were queried to ensure full adherence to accelerometer wearing. Immediately after removal of accelerometers from participants, data were downloaded and inspected visually for integrity (including 24 hours’ continuous data) using ActiLife software v6.13.3 (Actigraph, Pensacola, FL). ActiLife software converted the sample into 1Hz “counts” (one second epochs) for further analysis on subsequent programs. Counts represent the magnitude of acceleration after filtering to attenuate frequencies not associated with human movement. Data were then exported from the ActiLife software into a custom MATLAB program (Mathworks; Natick, MA) to calculate several previously reported metrics. The thresholding method reported by Urbin and Lang [35, 36] was used to determine if movement was present in each 1 second epoch. Total duration of movement of each limb was calculated, as well as duration of unilateral and bilateral movements. Separation of movement into unilateral only or simultaneous bilateral was possible since the data streams from the two accelerometers were synchronized. We calculated use ratios (activity ratio of hours of movement of the paretic limb to the non-paretic limb) [37–39] account for differences in overall activity levels across participants. The analysis code is available upon reasonable request. Accelerometry data includes any physical (48% of the sample) or occupational therapy (52% of sample) received. Nearly all therapy sessions were initial assessments. Therapeutic intervention, when it occurred, was directed at ADL oriented compensatory strategies and not at motor restoration [7, 9, 40] of the paretic UE.

Descriptive statistics on participant demographics, clinical function tests, and accelerometry were calculated. Pearson’s correlations were computed between clinical function tests (UEFM, ARAT, and SAFE score) and the accelerometry measures of movement count and use ratio on the paretic side in the stroke group to determine strength of relationship between these measures. Bonferroni corrections were applied for multiple pairwise comparisons (adjusted *p* = 0.01, for five pairwise comparisons). Correlations showed non-significant relationships between the clinical function tests and accelerometry measures, prompting an unplanned, principal component factor analysis on these measures to determine if they measured similar or different constructs. The number of components to extract was decided using Kaiser’s Eigenvalue greater than 1 criteria [41, 42]. A varimax orthogonal rotation was applied to improve interpretability of extracted factors. Factor loadings >0.4 were considered significant [43]. All analyses were completed using IBM SPSS Statistics, Version 25.

## Results

Twenty-five participants were recruited at 4.5 ± 1.8 days after stroke onset (see Table 1). Stroke severity ranged from very mild to moderately severe (total NIHSS 0–19, median = 37, IQR (0, 46)), and arm motor impairment was similarly distributed with UEFM scores ranging from 0–65 (median = 42, IQR (17,56)). Of the 25 participants; 8 had neglect and 8 reported mild to moderate pain. For controls, we recruited twelve acute rehabilitation inpatients (4 orthopedic, 5 leg amputation, 2 general debility and 1 cardiac). They had no neurological conditions and normal UE strength, sensation, and ROM on occupational therapy evaluation.

**Table 1:** Participant characteristics.

Demography		Side Affected by Stroke		
	Controls	All Stroke	Dominant	Non-Dominant
	n = 12	n = 25	n = 10	n = 15
Age	61 (12.7) 33–85	58 (13.4) 33–85	62.5 (13.3) 48–85	57.8 (15.1) 33–85
% Female	66.6	36	50	26.7
Days after onset	7.6 (2.1) 6–13	4.5 (1.8) 1–7	4.7 (2.0) 1–7	4.5 (1.7) 1–7
ARAT	Maximum Score = 57	27.8 (22.6)	28.3 0–57	27.5 (23.7) 0–57
UEFM	Maximum Score = 66	35.4 (22.2) 0–65	34.4 (22.6) 0–65	36.1 (22.8) 6–64
SAFE Score	9.5 (.52) 9–10	5.7 (3.5) 0–10	5.7 (3.5) 0–10	5.7 (3.7) 0–10
Active wrist extension, degrees	__	22.8 (18.1) 0–58	22.4 (18.1) 0–48	23.1 (18.1) 0–58
NIHSS Total Score	__	6.2(4.7) 0–19	6.5 (5.5) 0–19	6.0 (4.3) 0–16
NIHSS Motor Arm Score	__	1.5 (1.7) 0–4	1.4 (1.3) 0–4	1.6 (4.3) 0–4

Demographics of study participants. Values are mean (SD) with ranges underneath. Hand dominance was confirmed with patient or family. For between group comparisons, the dominant UE in control group was compared to non-paretic side in the stroke group, and non-dominant UE in controls with paretic side in stroke group, consistent with prior reports [13, 17, 34, 44–46].

**Fig 1:**
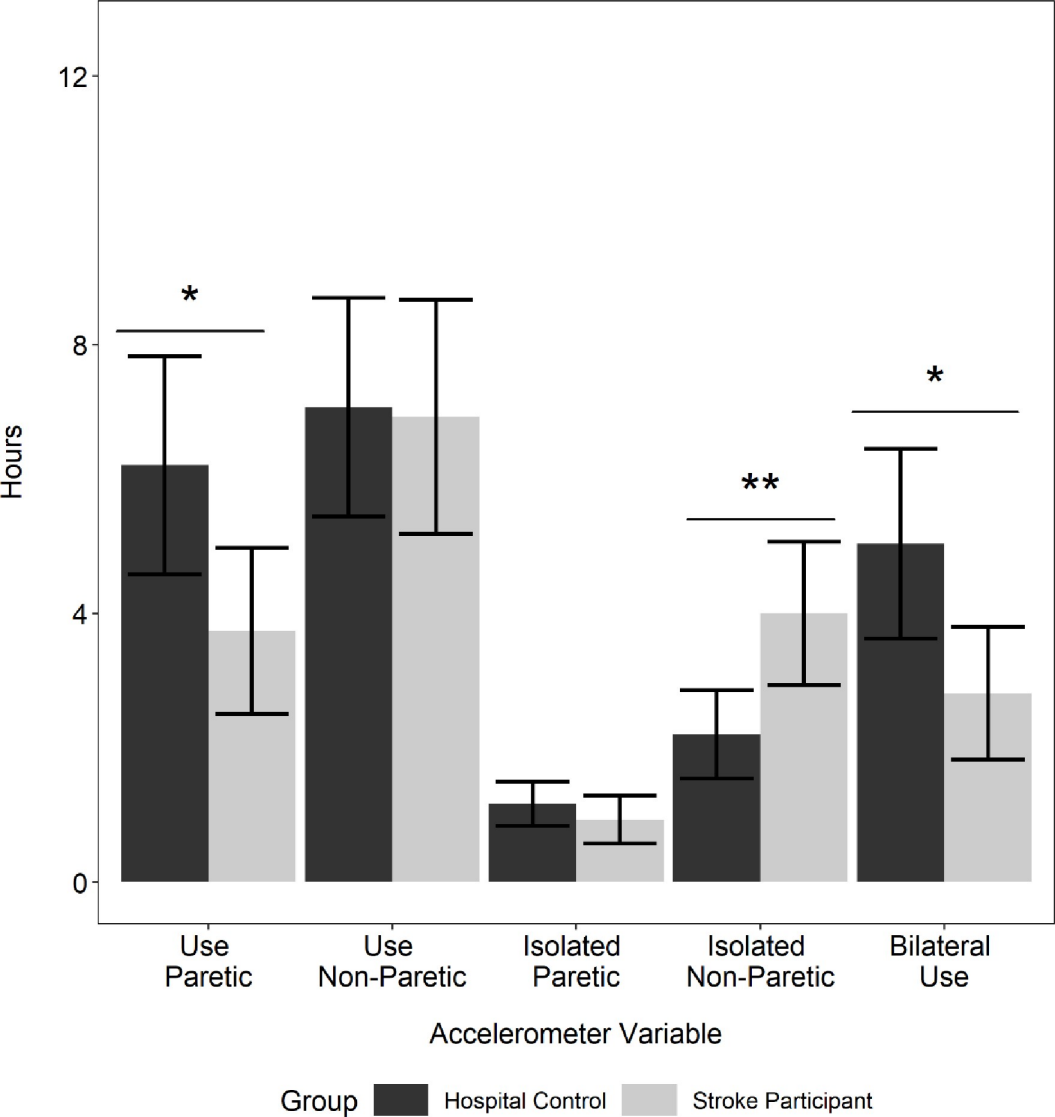
Quantification of UE movement using wrist-worn accelerometers. Stroke participants are compared to hospitalized controls. In Control participants (n = 12), the non-dominant limb was treated as the “paretic limb” and the dominant limb was treated as the non-paretic limb in those with stroke. Persons with stroke (n = 25) show a shift to unimanual use of the less affected side when compared to hospitalized controls, and significantly decrease their bilateral movements compared to hospitalized controls.

The amount of spontaneous UE movement as measured by accelerometry is presented in Table 2 and Fig 1. As in other published reports, we used the non-dominant UE activity of the control group as a benchmark [13, 17, 34, 44–46] for paretic limb activity. Overall, this sample of stroke participants showed considerable movement (3.7 ± 3.1 hours) of the paretic UE, that was nonetheless significantly reduced compared to controls’ non-dominant side (6.2 ± 2.8 hours; *t* (35) = -2.29, *p* = 0.03, Cohen’s d (-0.80). There was no significant difference between the amount of non-paretic UE movement in the stroke group compared to the dominant limb of controls (t (35) = -0.10, *p* = 0.92, Cohen’s d (-0.04). This amount of UE movement contrasts with community-based samples which generally display 3–6 hours of movement per limb [13, 17, 38, 45–50].

**Table 2:** Accelerometry data.

	Control ^+^ n = 12	All Stroke n = 25	Dominant Affected n = 10	Non-Dominant Affected n = 15	Comparison stroke to control p-value
Total UE ^#^ movement, paretic	6.2 ± 2.8	3.7 ± 3.1	4.4 ± 3.9	3.0+ 2.3	0.03 *
Total UE ^#^ movement, Non-paretic	7.0 ± 2.5	6.9 ± 4.5	8.2 ± 5.7	6.1 ± 3.3	0.92
Simultaneous Bilateral Movement	5.0 ± 2.5	2.8 ± 2.5	3.7 ± 3.3	2.2 ± 1.7	0.02 *
Paretic Unilateral	1.1 ± .60	0.93 ± .90	1.0 ± .79	.9 ± 1.0	0.41
Non-Paretic Unilateral	2.2 ± 1.2	3.9 ± 2.7	4.3 ± 3.4	3.7 ± 2.2	0.008 **
Use Ratio	.89 ± .20	.56 ± .30	.58 ± .30	.56 ± .37	0.001 **

Units are in Hours of Movement per 24 hours.

^†^In Control participants, the non-dominant limb was treated as the “paretic limb.”

^#^Total includes both unilateral and bilateral movement

* Significant at p ≤ 0.05

** significant at p ≤ 0.001. Reported p values for comparison between Control and Stroke groups

To investigate motor compensation patterns in participants within 4.5 days post stroke, we examined whether they compensated for hemiparesis by increasing bilateral UE movements or by increasing unilateral movements of the non-paretic UE [10, 11]. Comparison of unilateral use of the non-paretic UE in participants with stroke (3.9 ± 2.7 hours) and dominant UE in controls (2.2 ± 1.2 hours) showed a significant increase of the amount of movement in the non-paretic UE in stroke patients (*t* (34.7) = 2.81, *p* = 0.008, Cohen’s d (0.77)). Thus, participants with stroke increased the unilateral use of their non-paretic limb (3.9 ± 2.7 hours) compared to controls (2.2 ± 1.2 hours). Moreover, there was a significant reduction of bilateral UE use in stroke (2.8 ± 2.5 hours) compared to controls (5.0 ± 2.5 hours; (*t* (21.98) = -2.532, *p* = 0.02, Cohen’s d (-0.89)). The use ratio in stroke participants (0.56 ± 0.34) was significantly lower compared to the control participants (0.89 ± 0.18, *t* (34.3) = -3.79, *p* = 0.001, Cohen’s d (-1.10)). Taken together these data indicate increased unilateral use of the non-paretic UE for compensation.

Next, we examined the relationship between UE motor function measured with performance scales and the amount of spontaneous movement measured with accelerometry. The correlation matrix (Table 3) shows strong significant correlations between UEFM, ARAT, SAFE, and NIHSS motor arm item (Bonferroni adjusted *p* = 0.01). Use ratio was moderately correlated with each of the performance scales. Notably, there was no significant difference in the paretic arm hours of movement (t-1.54 (22), p = 0.14) in those with a high or low UEFM scores determined using a median split (high ≥33, low ≤32). Thus, in our sample assessed early after stroke, individuals with low UEFM scores moved their UE’s just as much as those with high UEFM scores who are often predicted to have better long-term recovery [39, 51, 52]. This dissociation between performance scales and amount of spontaneous movement was an unexpected finding that suggested that these might measure different dimensions of UE impairment. To test this hypothesis, we computed a principal component factor analysis using an orthogonal (Varimax) rotation to UEFM, ARAT, SAFE scores, range of motion at wrist, range of motion at metacarpo-phalangeal joint, and hours of movement from paretic and non-paretic side, see Table 4.

**Table 3:** Correlation matrix between clinical function tests and accelerometry-derived measures in Stroke group.

	ARAT	UEFM	SAFE	NIHSS Motor Arm	Use Ratio	Paretic Limb Use (Hrs)
ARAT	1					
UEFM	.097 **	1				
SAFE	0.88 **	0.93 **	1			
NIHSS Motor Arm	-0.85 **	-0.87 **	-0.89 **	1.00		
Use Ratio	0.65 **	0.69 **	-0.69 **	-0.69 **	1	
Paretic Limb Use (Hrs)	0.32	0.34	0.49	-0.48	0.51 **	1

**Correlation is significant at 0.01 level, adjusted *p* for multiple comparisons (5 pairwise comparisons, original *p* = 0.05).

**Table 4:** Item loadings based on two-factor solution with oblique rotation.

	Component	
	1 (Motor Performance)	2 (Quality of Movement)
Non-Paretic Limb Use (Hrs)	-0.18	0.95
Paretic Limb Use (Hrs)	0.34	0.09
ARAT	0.96	-0.12
SAFE	0.97	0.17
UEFM	0.99	-0.02
Wrist Extension ROM	0.95	-0.10
NIHSS Motor Arm	-0.92	-0.18

Components 1 and 2 explain 92.8% of the variability in UE impairment measured by the combination of variables entered into the factor analysis. Hours of use of paretic and non-paretic sides load on Component 2 (“Quantity of movement”), suggesting these measures represent a distinct construct compared to variables loading on Component 1 (“Motor performance”).

The final rotated solution achieved a simple structure and revealed a two-factor solution, explaining 92.8% of the variability in UE impairment. Component 1 showed high factor loadings from UEFM, ARAT, SAFE, and NIHSS scales with range of movement (wrist extension). Given the predominance of variables assessing movement performance on this component, we labeled Component 1 as aligning with the latent construct of “Motor performance”. Hours of movement of the paretic and non-paretic UE load heavily on Component 2. Given that both these accelerometry measures align with the amount of movement, we labeled Component 2 as aligning with the latent construct of “Quantity of movement”. Overall, 92.8% of the variance of UE movement in the first week after stroke was explained by the sum of Motor performance factor (67.4%) and by the Quantity of movement (25.4%).

## Discussion

We quantified the amount of paretic and non-paretic UE movement in acute stroke patients (mean = 4.5 ± 1.8 days). Prior studies have reported accelerometry outcomes in chronic stroke, at least 6 months or later [13, 17, 35, 45–47, 50, 53] we extend prior findings to acute stroke by demonstrating that even in the early days after stroke, individuals shift UE movements to the unaffected side rather than use a bimanual strategy. Additionally, accelerometry measures a distinct construct “quantity” of movement, independent of “quality” of movement measured by clinical function tests like ARAT and UEFM. Trialists using accelerometry to quantify UE motor function need to be cautious about interpreting accelerometry counts as a proxy for change in the quality of motor function after stroke in the absence of clinical function tests like the UEFM or ARAT.

Our study is novel in that it examines poorly characterized motor behaviors in the interval between stroke onset and typical rehabilitation interventions. Animal models of stroke recovery demonstrate patterns of gene expression and injury responses in the first few days after injury that evolve with time [54–57]. In animals, these processes might constitute a time-limited “sensitive period” for enhanced recovery after stroke [58, 59]. In this pilot study of motor behaviors during the first week after stroke, we characterized spontaneous motor behaviors in patients [60, 61]. Clinically, motor activity during the first weeks after stroke is poorly understood, because it falls outside the early intervention window of interest to vascular neurologists and before the substantive interventions of interest to rehabilitation investigators. We found several notable features.

First, motor performance measures are strongly correlated at this early post-stroke time-point, just as they have been demonstrated to correlate at later times after stroke [39, 47, 51, 62–64]. Assuming this correlation continues during recovery, this finding has the potential to simplify future studies during this time period, allowing a leaner UE assessment with fewer motor performance measures, reducing subject burden.

Second, we found that participants used movement compensations associated with relatively poor motor recovery in animal models of stroke. The adoption of compensatory (1) non-use of the impaired limb and simultaneous (16); (2) increased motor activity of the non-impaired limb is an important finding, particularly when it occurs within 4.5 days post stroke on average. It is widely accepted that individuals with stroke use compensatory movements [65–69]; our data are the first to reliably quantify the specific compensatory strategies adopted by participants at this early time. These compensations are especially important for neurorehabilitation because animal models of recovery have consistently demonstrated that (1) the overuse of the affected forelimb results in learned non-use and (2) motor practice with the non-paretic forelimb, or skill acquisition in the non-paretic forelimb inhibits recovery of the paretic forelimb [10, 11]. While we cannot claim to have measured learned non-use in this study,(16) the non-use we did observe could contribute to learned non-use over time. The compensations we identified in stroke participants suggest some of the maladaptive mechanisms may already be set in motion within the first week after stroke. If interpreted in the context of animal models of stroke recovery, accelerometry measures are an important covariate of motor recovery that trialists need to control for, particularly because they affect recovery, and measure a different construct not captured by routinely used clinical motor performance measures like the UEFM and ARAT. Further supporting the picture of non-paretic limb compensation, the amount of bilateral simultaneous movements was significantly reduced compared to controls. Thus bilateral movements were not the preferred compensation strategy in our sample.

Finally, the factor analysis identified two latent constructs underlying measurement of UE impairment. Component 1 aligned with “Motor performance’ and Component 2 with “Quantity of movement”. Motor performance, measured by a combination of clinical function tests like UEFM, ARAT, and SAFE accounts for 67% of the variance in UE impairment; whereas Quantity of movement, measured by accelerometry, accounts for an additional 25% of the variance. This two-factor solution is a parsimonious solution as evidenced by the amounts of variance explained by each factor. A third component only added an additional 2% variance in UE impairment compared, suggesting the 2-factor solution provided the optimal solution. The orthogonal rotation resulted in motor performance measures aligning to Component 1 and accelerometry measures aligning to Component 2. Although it is non-traditional for a “factor” to have only two variables with significant loadings as in the case of our Component 2, this two-factor solution is statistically and clinically meaningful. Together, the different metrics we used could account for approximately 93% of the variance observed in UE impairment within a week after stroke. These results highlight the need to measure both motor performance and quantity of movement when evaluating UE function during this post-stroke period.

Our work extends that of Gebruers et al. [70] and others [71]. Our results are generally consistent with their correlations between use ratio and UEFM. However, we did not find a correlation between hours of movement and UEFM. The differences arise because we used Pearson’s correlation, given the non-parametric nature of UEFM. When we tested correlations between hours of movement and UEFM using Spearman’s test, our findings were consistent with Gebruers et al. [52].

Importantly our approach explains why Gebruers et al. [70] found that hours of movement were not good predictors of disability at 3-months. Gebruers et al. found hours of motion were a good predictor only when there was going to be a "good outcome", not if there was a "bad outcome" at 3 months. Our factor analysis explains this dissociation between the good outcome and bad outcome groups: hours of movement does not measure the same construct as UEFM. In individuals with high UEFM scores, hours of movement are highly correlated with performance measures. However, in participants with low UEFM scores, there is only a weak correlation with hours of movement, providing a poor prediction of outcome at 3 months. Our analysis emphasizes this dissociation; there is no significant difference between hours of movement in those with high versus low UEM scores. Thus, measuring both hours of movement and motor performance is required to realistically assess motor function. Use ratios are less affected, because they are loaded on the motor performance axis, similar to the UEFM. Trialists considering accelerometry should interpret counts cautiously, particularly because they do not measure the same construct as quality of motor performance.

Our prospectively designed study is important and novel in that it is a probe into how individuals with stroke spontaneously adapt UE movements and adopt compensatory motor strategies in the early days after stroke. In rodent models, intense non-paretic forelimb skill training has been shown to inhibit motor recovery of the paretic forelimb [10, 11]. In stroke participants, a 56% increase in isolated unimanual use of the non-paretic UE is certainly striking. How this increase compares to unimanual training models in animals is unknown, because the spontaneous behaviors exhibited by our participants are quite different in concept from the structured high-intensity repetitive training used in the animal models that motivate our study. Further research is needed to discern whether in human clinical populations early after stroke modification of spontaneous motor behaviors might positively (or even negatively) affect eventual motor outcome.

### Study limitations

The sample recruited from an urban safety net hospital may not be representative of all stroke patients, and future studies should recruit a larger and more representative sample. The cross-sectional design and 24-hour assessment period do not allow assessment of the rapid motor changes seen in the first days of stroke. Our attempts to collect 30-day outcome data were hampered by participant unavailability, death or recurrent stroke, and lack of recall of study consent by some participants and families. Other constructs relevant to UE behavior may exist.

## Conclusion

With this study we were successful in recruiting and testing people with stroke within one week of onset. The sample ranged from very mild to severely impaired participants as measured by highly correlated motor performance measures. Accelerometry metrics in these early days after stroke show an overall reduction of bilateral UE movement with individuals compensating by shifting their UE movement to the non-paretic limb. Compensatory strategies chosen by patients provide the substrate for potential detrimental behaviors discovered in animal recovery models: learned non- use and inhibition of motor recovery by training of the unimpaired forelimb. Finally, factor analysis shows that assessment of motor performance alone is insufficient to describe UE movement, adding accelerometry explains 93% of the variance completing the assessment of UE behavior early after stroke.
